# Supplementary material for: LUBAC modulates CBM complex functions downstream of TRAF6 in T cells
Source: Nat Commun. 2025 Nov 10;16:9899. doi: 10.1038/s41467-025-65879-6 (PMC12602705; doi:10.1038/s41467-025-65879-6)
Supplement: Supplementary file 1 — Supplementary Information [file 41467_2025_65879_MOESM1_ESM.pdf]

Supplementary Information for

LUBAC modulates CBM complex functions downstream of TRAF6 in T cells

Carina Graß, Franziska Ober, Daniel Krappmann et al

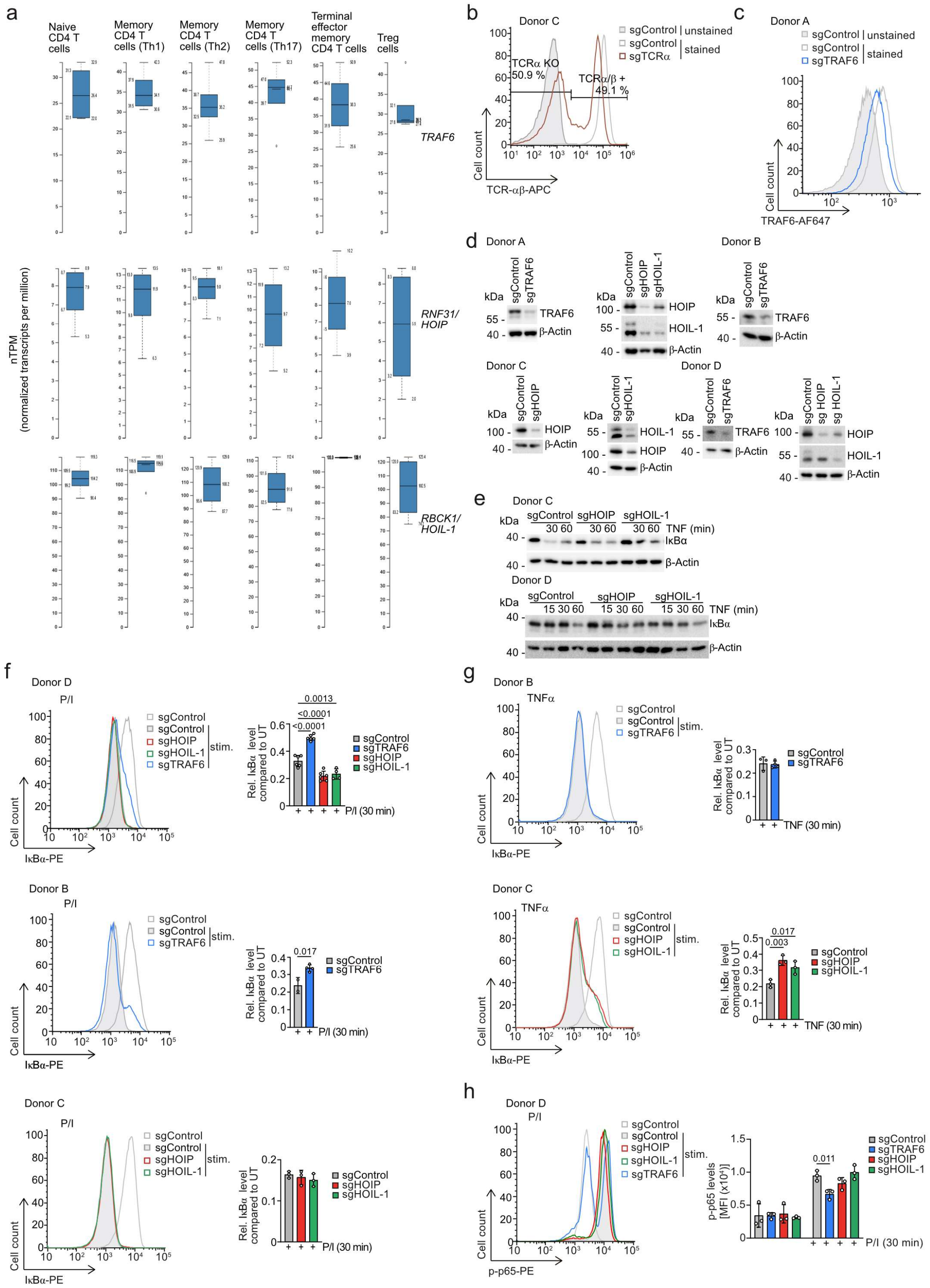

Supplementary Figure 1: Analyses of LUBAC and TRAF6 KO in human CD4 T cells. (a) Expression of *TRAF6*, *RNF31/HOIP* and *RBCK1/HOIL-1* transcripts in human CD4 T cell subsets from PBMCs of healthy donors. **(b)** Histogram of flow cytometry depicting KO efficiency using sgTCRα targeting and staining of TCRα/β receptor on human CD4 T cells. **(c)** TRAF6 protein reduction analyzed by flow cytometry after sgTRAF6 transfection into human CD4 T cells. **(d)** Western blot analyses showing KO in different donors after sgControl, sgTRAF6, sgHOIP and sgHOIL-1 transfection into primary CD4 T cells. **(e)** IκBα Western blot analysis of sgControl, sgHOIP and sgHOIL-1 primary CD4 T cells after stimulation with TNF for the indicated time points. **(f, g)** Representative flow cytometric analyses (left panels) and quantification of changes in mean fluorescence intensity (MFI) values normalized to untreated [UT] (right panels) IκBα levels in sgControl, sgTRAF6, sgHOIP and sgHOIL-1 KO primary

CD4 T cells after P/I (f) or TNF (g) stimulation (30 min) for different donors. Number of biological replicates relied on availability of T cells from individual donors. Donor D: n=6; n=4 for sgHOIL-1, donor B (n=2 sgControl, n=4 sgTRAF6, n=3 in g), donor C (n=3 in f and g). IκBα levels of UT samples were set to 1. Treated samples were normalized to the untreated samples. **(h)** Representative flow cytometric analyses (left panel) and quantification of changes in MFI (right panel) of p-p65 levels in sgControl, sgTRAF6, sgHOIP and sgHOIL-1 primary CD4 T cells after P/I stimulation (30 min) shown for donor D from 3 biological replicates. All bars show the means ± SD and p-values were calculated by unpaired student t-test (donor B in f, g), one-way ANOVA (donor C and D in f, g) or two-way ANOVA (h) combined with Dunnett's multiple comparisons test. Only significant p-values (<0.05) are shown. kDa: kilodalton.

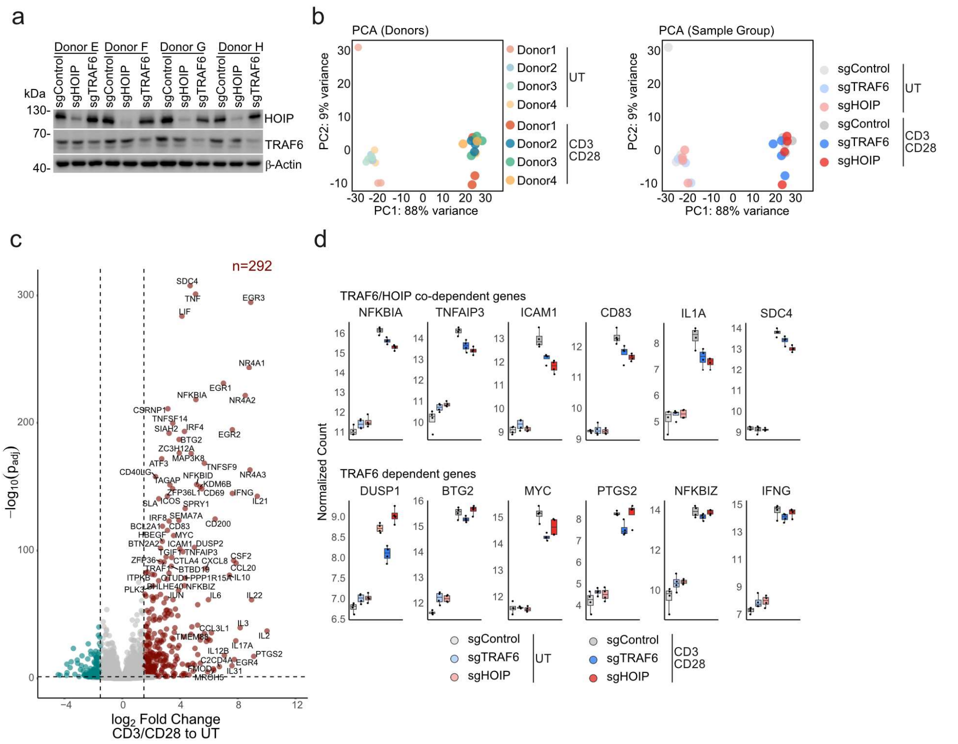

Supplementary Figure 2: Transcriptomic analyses in TRAF6 and HOIP KO CD4 T cells. **(a)** Western Blot analysis showing KO in donor E-H after transfection with sgControl, sgTRAF6 or sgHOIP. **(b)** Principal Component Analysis (PCA) of the RNAseq data, highlighting donors and sample groups. (**c**) Volcano plot showing the differentially expressed genes (|log2FoldChange|≥1.5 & p_{adj}≤0.05) upon CD3/CD28 stimulation compared to untreated control. Up- and down-regulated genes upon stimulation are highlighted in red and blue, respectively. (**d**) Boxplots of TRAF6- and HOIP-dependent gene expression. The interquartile range (IQR) around the median was represented, the whiskers extend to 1.5x IQR from the quartiles. Dependency analysis as described in Methods. kDa: kilodalton.

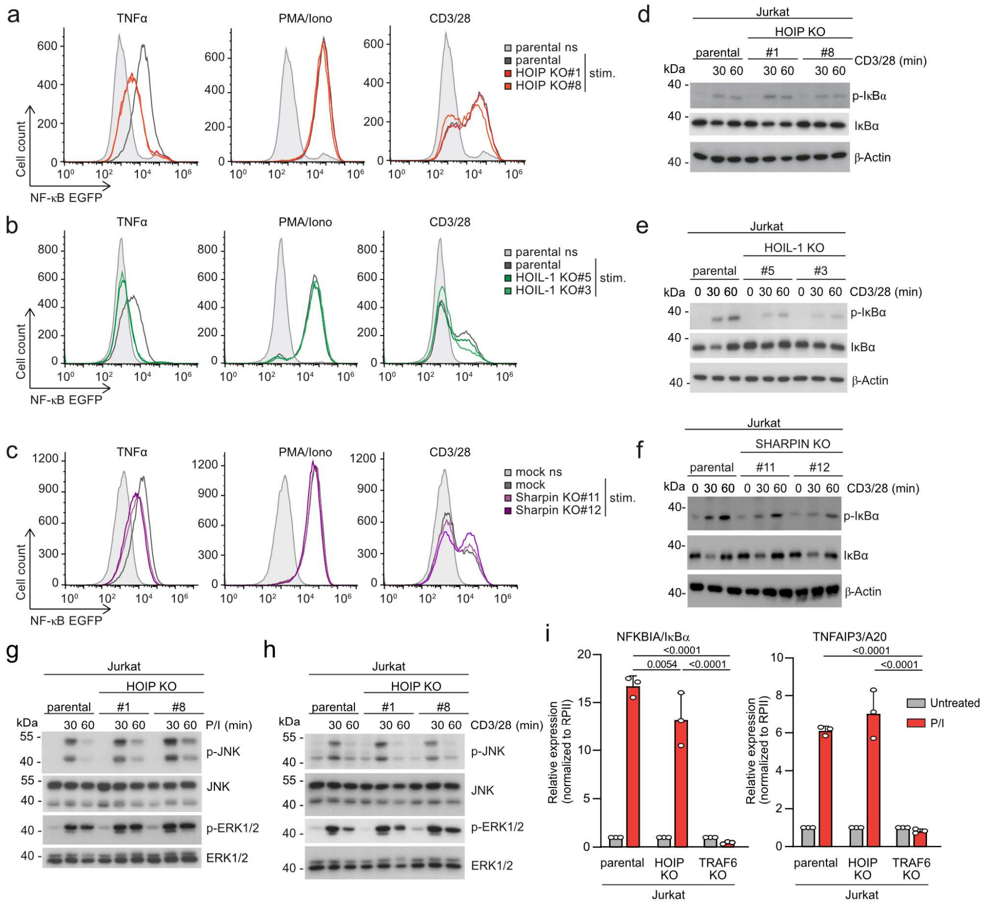

Supplementary Figure 3: Analyses of LUBAC KO Jurkat T cells. (a-c) Representative flow cytometric analyses of NF-κB EGFP reporter in parental and LUBAC KO Jurkat T cell clones after TNF, P/I and CD3/28 stimulation for 5 h. **(d-f)** NF-κB signaling was analyzed in parental and KO Jurkat T cells after stimulation with CD3/CD28 for the indicated time points. Western blots showing IκBα phosphorylation and degradation are shown. **(g, h)** Activation/phosphorylation of JNK and ERK was assessed on Western blot in parental and HOIP KO Jurkat T cells upon P/I (g) and CD3/28 (h) stimulation. **(i)** RT-PCR analyses of NFKBIA/IκBα and TNFAIP3/A20 mRNA in WT, HOIP KO, and TRAF6 KO Jurkat T cells following P/I stimulation for 1 hour was determined from 3 biological replicates. All bars show the means ±SD, and p-values were calculated by two-way ANOVA combined with Tukey's multiple comparison test. Only significant p-values (<0.05) are shown. kDa: kilodalton.

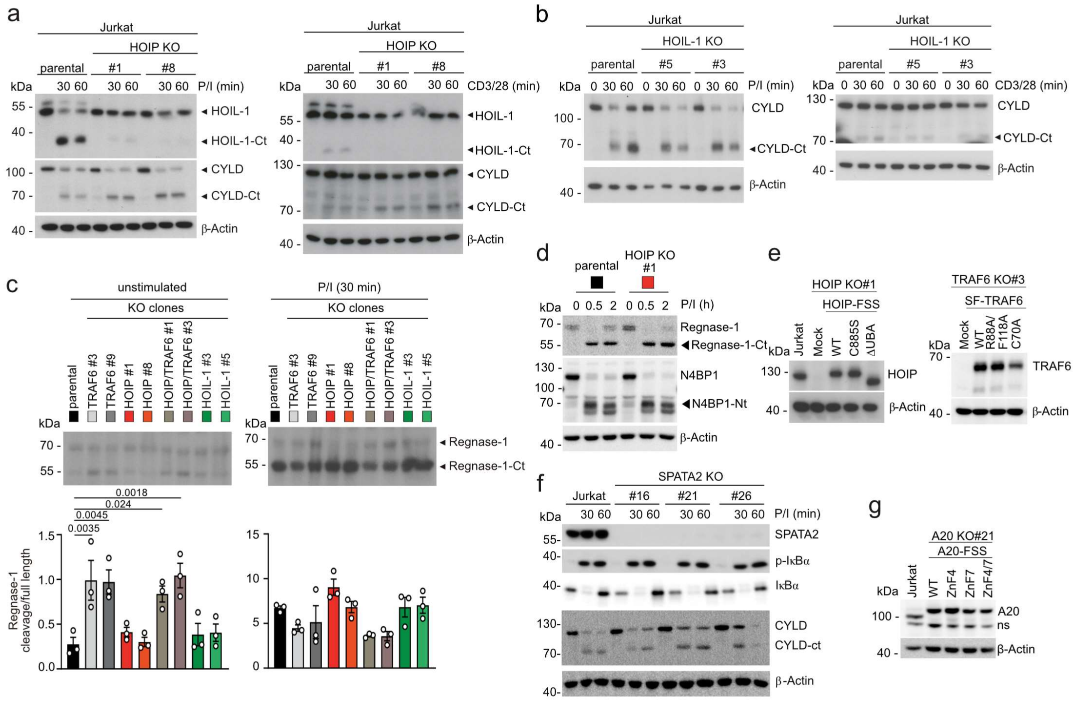

Supplementary Figure 4: Modulation of substrate recognition by TRAF6 and LUBAC. **(a, b)** Parental and HOIP KO (a) or HOIL-1 KO (b) Jurkat cells were stimulated with P/I or CD3/CD28 for the indicated time points and MALT1 substrate cleavage of CYLD and HOIL-1 was detected by Western blot. **(c)** Cleavage of Regnase-1 was detected in parental, as well as two independent clones of TRAF6 KO, HOIP KO, TRAF6/HOIP DKO or HOIL-1 KO Jurkat T cells either untreated or P/I stimulated (30 min) by Western blot. For quantification, the ratios of cleavage products to full-length proteins were determined from 3 biological replicates. All bars represent the means ±SEM and p-values were calculated by one-way ANOVA combined with Dunnett's multiple comparisons test. Only significant p-values (<0.05) are shown. **(d)** Analyses of Regnase-1 and N4BP1 cleavage in parental and HOIP KO Jurkat T cells after 0.5 and 2 h P/I stimulation. **(e)** Parental HOIP KO Jurkat cells reconstituted with either HOIP WT-FSS, HOIP C885S-FSS or HOIP ΔUBA-FSS via lentiviral transduction. Western blot of TRAF6 KO Jurkat cells transduced with either SF-TRAF6 WT, SF-TRAF6 R88A/F118A or SF-TRAF C70A. **(f)** IκBα phosphorylation and degradation and CYLD cleavage was assessed in three independent SPATA2 KO clones after P/I stimulation by Western blot. **(g)** Western blot of A20 KO Jurkat cells reconstituted with A20-FSS tag WT or ZnF4, ZnF7, ZnF4/7 mutants via lentiviral transduction. kDa: kilodalton.

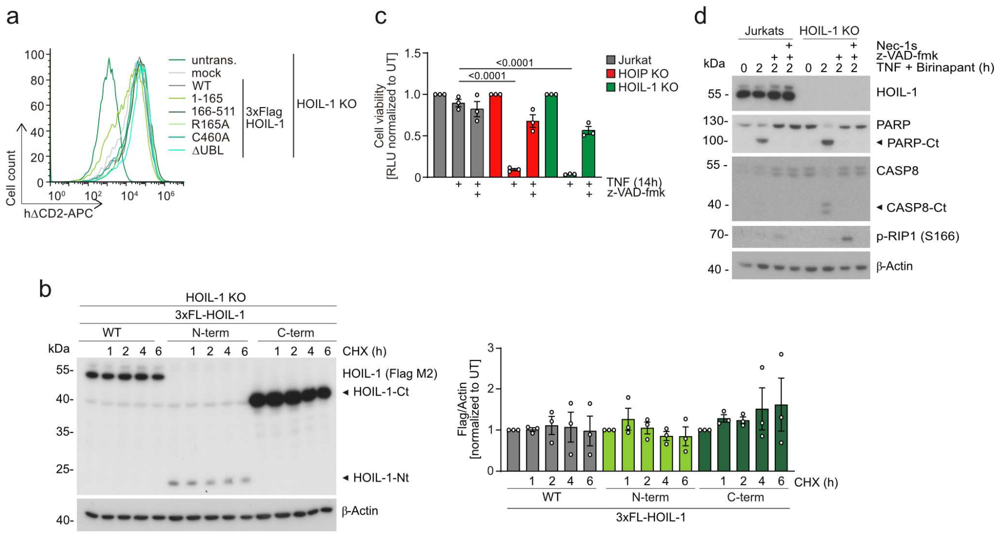

Supplementary Figure 5: Stability of HOIL-1 cleavage fragments and LUBAC-dependent cell death. (a) Transduction of 3x-Flag-tagged HOIL-1 WT and mutants in HOIL-1 KO NF-κB-EGFP Jurkat T cells was monitored by surface-marker hΔCD2 in flow cytometry. **(b)** Stability of HOIL-1 WT, N-term and C-term in HOIL-1 KO Jurkat T cells was assessed after cycloheximide (CHX) chase experiments at various time points by Western blot. For quantification, HOIL-1 bands were normalized to β-Actin, and values for untreated samples were set to 1. **(c)** Cell viability determined by CellTiter-Glo assay was determined after 14 h TNF or TNF+zVAD-FMK treatment in parental, HOIP KO and HOIL-1 KO Jurkat T cells from 3 biological replicates. Relative luminescence units (RLU) values were normalized to untreated (UT) samples of each cell line. **(d)** Parental and HOIL-1 KO Jurkat T cells were treated with TNF/Birinapant (IAP degrader) in the absence or presence of zVAD-FMK (inhibition apoptosis) or zVAD-FMK/Nec1-s (inhibition apoptosis and necroptosis) for 2 h. PARP and CASP8 cleavage as well RIP1 S166 phosphorylation was analyzed by Western blot. All bars show the means ± SEM and p-values were calculated by one-way ANOVA combined with Tukey's (b) or Dunnett's (c) multiple comparisons test. Only significant p-values between treated samples are shown. kDa: kilodalton.

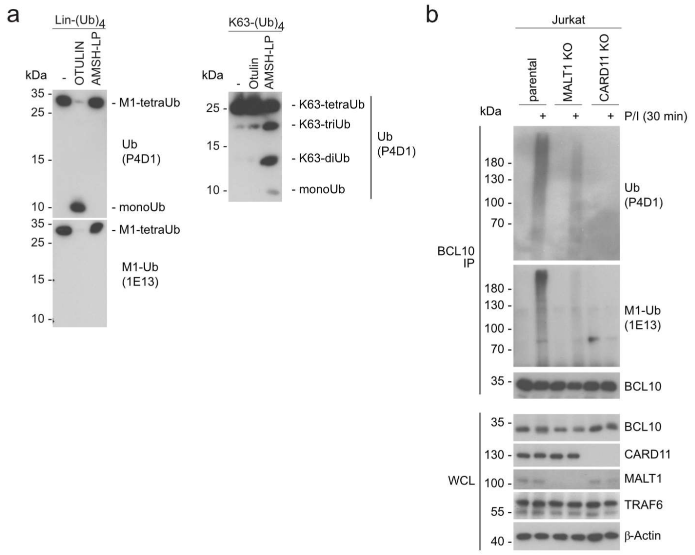

Supplementary Figure 6: Analyses of BCL10 ubiquitination. (a) In vitro cleavage assay to determine linkage specificity of BCL10 ubiquitination. M1- or K63-linked tetra-ubiquitin chains were incubated with OTULIN or AMSH for 60 min at 30°C. **(b)** Parental, MALT1 KO or CARD11 KO Jurkat T cells were stimulated with P/I for 30 min. After denaturing lysis, immunoprecipitation (IP) using anti-BCL10 antibody was performed and BCL10 modifications were detected by Western Blot using total ubiquitin (P4D1) or M1-ubiquitin (1E13) antibodies. kDa: kilodalton.

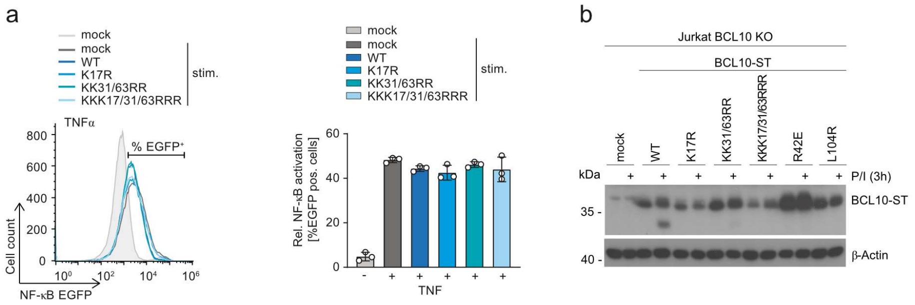

Supplementary Figure 7: Functional impact of potential ubiquitin acceptor sites in BCL10. (a) NF-κB-EGFP reporter induction of BCL10 KO Jurkat T cells reconstituted with BCL10 WT and mutants (K17R, KK31/63RR, KKK17/31/63RRR) after TNF stimulation (5 h) was analyzed by flow cytometry. Representative flow cytometry of EGFP expression is presented in the upper panel and EGFP expression was quantified determining EGFP positive cells from 3 biological replicates. Bars show the means ±SEM and p-values were calculated by one-way ANOVA combined with Dunnett's multiple comparisons test. **(b)** BCL10 expression and cleavage were assessed after 3 h P/I stimulation in BCL10 WT and mutants reconstituted BCL10 KO Jurkat T was assessed by Western blot. kDa: kilodalton.

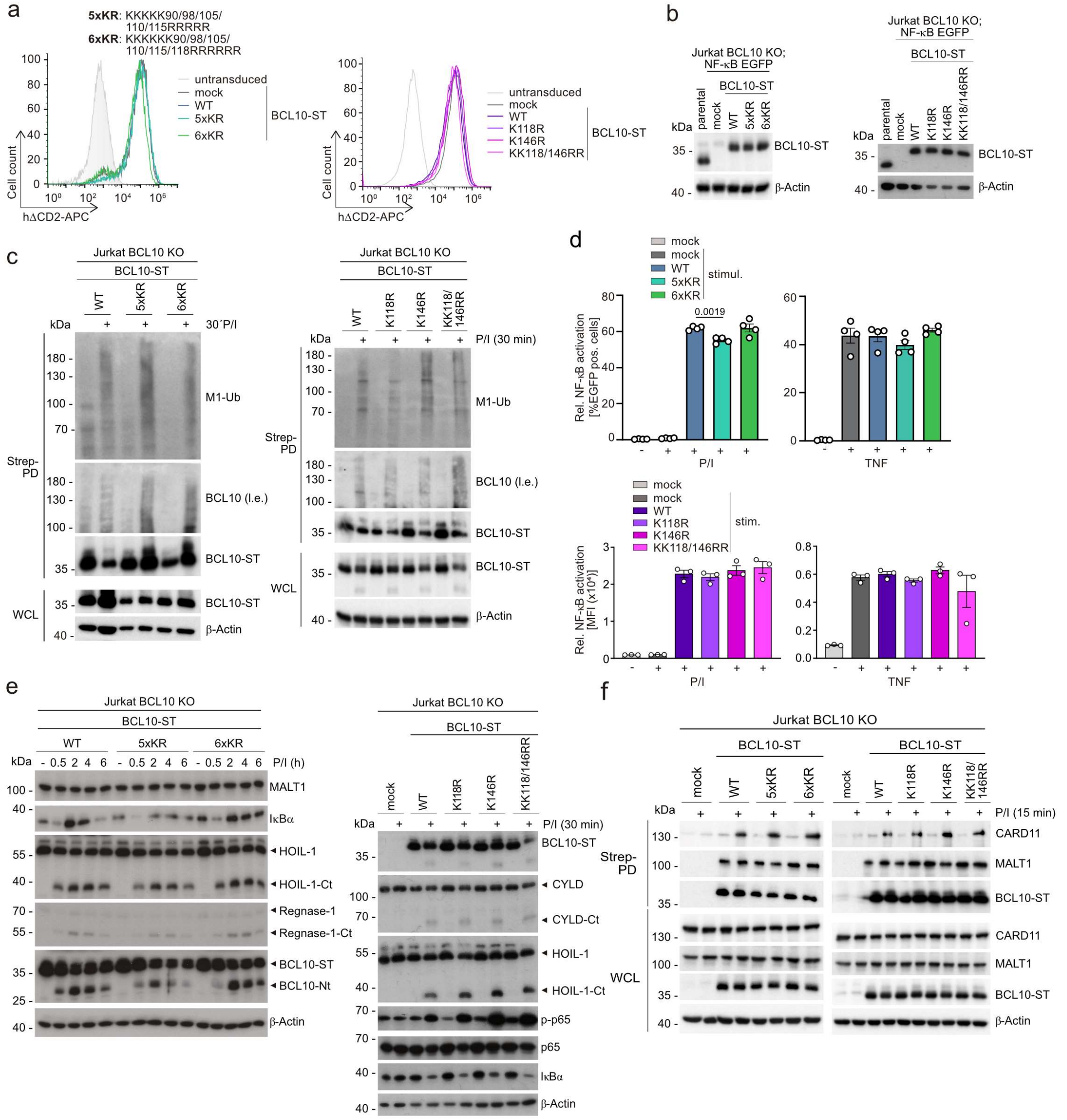

Supplementary Figure 8: Lack of impact of various lysine to arginine exchanges on BCL10 function. (a) Transduction of Strep-tagged (ST) BCL10 WT and 5xKR, 6xKR, K118R, K146R and KK118/146RR in BCL10 KO NF-κB-EGFP reporter Jurkat T cells was monitored using surface-marker hΔCD2 by flow cytometry. **(b)** Expression of BCL10 WT and KR mutants was analyzed by Western blot. **(c)** BCL10 KO Jurkat T cells reconstituted with ST-BCL10 WT and KR mutants were stimulated with P/I. After denaturing lysis, immunoprecipitation (IP) using Strep-Tactin pulldown (Strep-PD) was performed and BCL10 modifications were analyzed by Western blot using M1-ubiquitin (1E13) or BCL10 antibodies. **(d)** NF-κB-EGFP reporter induction of BCL10 KO Jurkat T cells reconstituted with BCL10 WT and KR mutants after P/I (left) or TNF stimulation (5 h) was analyzed by flow cytometry. EGFP expression was quantified determining number of EGFP positive cells from 4 biological replicates (upper panel) or by calculating the median fluorescence intensity (MFI) from 3 biological replicates (lower panel). Bars show the means ±SEM and p-values were calculated by one-way ANOVA combined with Dunnett's multiple comparisons test. Only significant p-values (<0.05) are shown. **(e)** NF-κB signaling (IκBα degradation) and MALT1 substrate cleavage (HOIL-1, Regnase-1, BCL10 and CYLD) were examined in BCL10 KO Jurkat T cells reconstituted with BCL10 WT and KR mutants after P/I stimulation. **(f)** BCL10 KO Jurkat T cells reconstituted with Strep-tagged (ST) BCL10 WT and KR mutants were stimulated with P/I. Strep-Tactin

pulldowns (Strep-PD) were performed and binding of BCL10-MALT1 and BCL10-CARD11 was analyzed by Western blot. kDa: kilodalton.

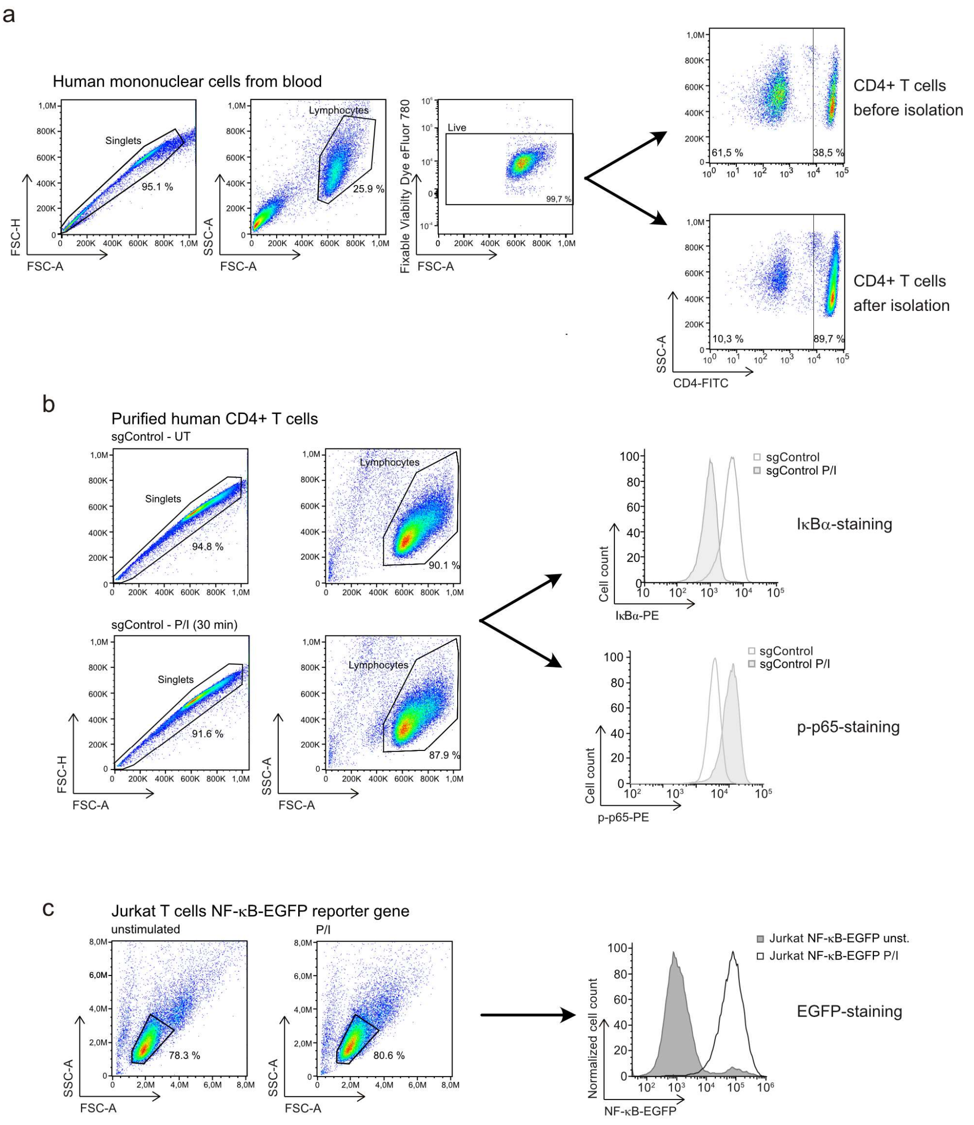

Supplementary Figure 9: Purification CD4$^+$ T cells and gating strategies. (a) Mononuclear cells from fresh blood were isolated using negative selection of CD4$^+$ T cells. Purification strategy efficacy is shown, which was used for all donors in Fig. 1, 2, and Supplementary Fig. 1, 2. (b) Exemplified strategy for IκBα and p-p65 staining on purified human CD4+ T cells used in Fig. 1c-e, and Supplementary Fig. S1f-h. The same strategy was used for TRAF6 (Fig. S1c) and TCR-α/β (Supplementary Fig. S1b) staining. (c) Exemplified strategy for staining of EGFP in Jurkat T cells transduced with NF-κB-EGFP reporter gene (pHAGE-Igκ(6x)cona-HygEGFP) used in Fig. 3d-f, 3k, 3m, 6c, 8d, and Supplementary Fig. 3a-c, 7a, 8d.

Supplementary Table 1: DNA constructs

DNA constructs	Source
pHAGE_hΔCD2-T2A-Mock-FS	(Schlauderer *et al*, 2018)
pHAGE_hΔCD2-T2A-BCL10 WT-FSS	(Schlauderer *et al.*, 2018)
pHAGE_hΔCD2-T2A-BCL10 R42E-FSS	(Schlauderer *et al.*, 2018)
pHAGE_hΔCD2-T2A-BCL10 L104R-FSS	(Schlauderer *et al.*, 2018)
pHAGE_hΔCD2-T2A-BCL10 K17R-FSS	This paper
pHAGE_hΔCD2-T2A-BCL10 KK31/63RR-FSS	This paper
pHAGE_hΔCD2-T2A-BCL10 KKK17/31/63RRR-FSS	This paper
pHAGE_hΔCD2-T2A-BCL10 5xKR (KKKKK90/98/105/110/115RRRRR)-FSS	This paper
pHAGE_hΔCD2-T2A-BCL10 6xKR (KKKKKK90/98/105/110/115/118RRRRRR)-FSS	This paper
pHAGE_hΔCD2-T2A-BCL10 K118R-FSS	This paper
pHAGE_hΔCD2-T2A-BCL10 K146R-FSS	This paper
pHAGE_hΔCD2-T2A-BCL10 KK118/146RR-FSS	This paper
pHAGE_hΔCD2-T2A-TRAF6 WT-FSS	(O'Neill *et al*, 2021)
pHAGE_hΔCD2-T2A-TRAF6 C70A-FSS	(O'Neill *et al.*, 2021)
pHAGE_hΔCD2-T2A-TRAF6 R88A/F118A-FSS	(O'Neill *et al.*, 2021)
pHAGE_hΔCD2-T2A-MALT1A WT-FSS	(O'Neill *et al.*, 2021)
pHAGE_hΔCD2-T2A-MALT1A E316A/E806A-FSS	(O'Neill *et al.*, 2021)
pHAGE_hΔCD2-T2A-HOIP WT-FSS	This paper
pHAGE_hΔCD2-T2A-HOIP C885S-FSS	This paper
pHAGE_hΔCD2-T2A-HOIP ΔUBA-FSS	This paper
pHAGE_hΔCD2-T2A-3xFL-HOIL-1 WT	This paper
pHAGE_hΔCD2-T2A-3xFL-HOIL-1 1-165	This paper
pHAGE_hΔCD2-T2A-3xFL-HOIL-1 166-511	This paper
pHAGE_hΔCD2-T2A-3xFL-HOIL-1 R165A	This paper
pHAGE_hΔCD2-T2A-3xFL-HOIL-1 C460A	This paper
pHAGE_hΔCD2-T2A-3xFL-HOIL-1 ΔUBL	This paper
pHAGE_hΔCD2-T2A-HOIL-1 WT-FSS	This paper
pHAGE_hΔCD2-T2A-HOIL-1 1-165-FSS	This paper
pHAGE_hΔCD2-T2A-HOIL-1 166-511-FSS	This paper
pHAGE-hΔCD2-T2A-A20-FSS	(Yin *et al*, 2022)
pHAGE-hΔCD2-T2A-A20ZnF4 mt-FSS (C624/627A)	This paper
pHAGE-hΔCD2-T2A-A20ZnF7 mt-FSS (C779/782A)	This paper
pHAGE-hΔCD2-T2A-A20ZnF4/7 mt-FSS (C624/627/779/782A)	(Yin *et al.*, 2022)
pEF HA-MALT1	(Oeckinghaus *et al*, 2007)
pEF HA-BCL10	(Schlauderer *et al.*, 2018)

pEF 3xFL mock	(Scharschmidt *et al*, 2004)
pEF 3xFL-BCL10 WT	(Wegener *et al*, 2006)
pEF 3xFL-BCL10 R42E	This paper
pEF 3xFL-BCL10 L104R	(Schlauderer *et al.*, 2018)
pEF 3xFL-BCL10 K17R	This paper
pEF 3xFL-BCL10 KK31/63RR	This paper
pEF 3xFL-BCL10 KKK17/31/63RRR	This paper
pHAGE-Igκ(6x)cona-HygEGFP	(Gehring *et al*, 2019)
pMD2.G	Addgene #12259
psPAX2	Addgene #12260
PX458	Addgene #48138
lentiCRISPRv2 vector	Addgene #52961

Supplementary Table 2: sgRNAs used for KO generation in Jurkat T cells

BCL10	5′AGTGAGGTCCTCCTCGGTGA 3′<br>5′TTCCGCTTTCGTCTCCCGCT 3′	(Schlauderer *et al.*, 2018)
TRAF6	5’-TGTTACAGCGCTACAGGAGC-3’<br>5’-ATGGTGAAATGTCCAAATGA-3’	(O'Neill *et al.*, 2021)
HOIP	5′-GAGAGCTGGCTAGTAGCGGC-3′	(Stangl *et al*, 2019)
HOIL-1	5′-ATGGACGAGAAGACCAAGAA-3′<br>5′-TGTACCACGATCTGGCACTG-3′)	(Yin *et al.*, 2022)
A20	5′-GAGGCAATTGCCGTCACCTG-3′	(Yin *et al.*, 2022)
SHARPIN	5´-GCTCTTGGCTGTGCACGCCG-3´<br>5´-GGGGACTGGGCAGGGAGACA-3´	This paper
SPATA2	5′-CGCAGGCACTCATCGCTGCC-3′	(Schlicher *et al*, 2016)
MALT1	5′-CCGTGGTCCAGATATATAGC-3′<br>5′-GGTTGAAGCAAATGCAATGC-3′	(Meininger *et al*, 2016)
CARD11	5′-CTCATCAATGACCTTACACTGACGCAGGTAGG-3′	(Seeholzer *et al*, 2018)

Supplementary Table 3: sgRNAs used for KO generation in primary CD4 T cells

TRAF6	5′ GAAGCAGTGCAAACGCCATG 3′ (ex2)	This paper
HOIP	5′ GCGATTATATGGCTACACAG 3′ (ex3)	This paper
HOIL-1	5′ GAGACGCCACTGTCATATCA 3′ (ex3)	This paper
TCRα	5′ ACAAAACTGTGCTAGACATG 3′ (ex1)	(O'Neill *et al.*, 2021)

Supplementary Table 4: Primer for RT-PCR

NFKBIA	5' AGGACGGGGACTCGTTCCTG 3 (forward)<br>5' CAAGTGGAGTGGAGTCTGCTG 3' (reverse)
TNFAIP3	5' CTGAAAACGAACGGTGACGG 3' (forward)<br>5' CGTGTGTCTGTTTCCTTGAGCG 3' (reverse):
RPII	5' GTTCGGAGTCCTGAGTCCGGATG 3' (forward)<br>5' CCTGCCTCGGGTCCATCAGC 3' (reverse)

Supplementary References

Gehring T, Erdmann T, Rahm M, Grass C, Flatley A, O'Neill TJ, Woods S, Meininger I, Karayel O, Kutzner K *et al* (2019) MALT1 Phosphorylation Controls Activation of T Lymphocytes and Survival of ABC-DLBCL Tumor Cells. *Cell Rep* 29: 873-888 e810

Meininger I, Griesbach RA, Hu D, Gehring T, Seeholzer T, Bertossi A, Kranich J, Oeckinghaus A, Eitelhuber AC, Greczmiel U *et al* (2016) Alternative splicing of MALT1 controls signalling and activation of CD4(+) T cells. *Nature communications* 7: 11292

O'Neill TJ, Seeholzer T, Gewies A, Gehring T, Giesert F, Hamp I, Grass C, Schmidt H, Kriegsmann K, Tofaute MJ *et al* (2021) TRAF6 prevents fatal inflammation by homeostatic suppression of MALT1 protease. *Sci Immunol* 6: eabh2095

Oeckinghaus A, Wegener E, Welteke V, Ferch U, Arslan SC, Ruland J, Scheidereit C, Krappmann D (2007) Malt1 ubiquitination triggers NF-kappaB signaling upon T-cell activation. *The EMBO journal* 26: 4634-4645

Scharschmidt E, Wegener E, Heissmeyer V, Rao A, Krappmann D (2004) Degradation of Bcl10 induced by T-cell activation negatively regulates NF-kappa B signaling. *Molecular and cellular biology* 24: 3860-3873

Schlauderer F, Seeholzer T, Desfosses A, Gehring T, Strauss M, Hopfner KP, Gutsche I, Krappmann D, Lammens K (2018) Molecular architecture and regulation of BCL10-MALT1 filaments. *Nature communications* 9: 4041

Schlicher L, Wissler M, Preiss F, Brauns-Schubert P, Jakob C, Dumit V, Borner C, Dengjel J, Maurer U (2016) SPATA2 promotes CYLD activity and regulates TNF-induced NF-κB signaling and cell death. *EMBO Rep* 17: 1485-1497

Seeholzer T, Kurz S, Schlauderer F, Woods S, Gehring T, Widmann S, Lammens K, Krappmann D (2018) BCL10-CARD11 Fusion Mimics an Active CARD11 Seed That Triggers Constitutive BCL10 Oligomerization and Lymphocyte Activation. *Frontiers in immunology* 9: 2695

Stangl A, Elliott PR, Pinto-Fernandez A, Bonham S, Harrison L, Schaub A, Kutzner K, Keusekotten K, Pfluger PT, El Oualid F *et al* (2019) Regulation of the endosomal SNX27-retromer by OTULIN. *Nature communications* 10: 4320

Wegener E, Oeckinghaus A, Papadopoulou N, Lavitas L, Schmidt-Supprian M, Ferch U, Mak TW, Ruland J, Heissmeyer V, Krappmann D (2006) Essential role for IkappaB kinase beta in remodeling Carma1-Bcl10-Malt1 complexes upon T cell activation. *Molecular cell* 23: 13-23

Yin H, Karayel O, Chao YY, Seeholzer T, Hamp I, Plettenburg O, Gehring T, Zielinski C, Mann M, Krappmann D (2022) A20 and ABIN-1 cooperate in balancing CBM complex-triggered NF-kappaB signaling in activated T cells. *Cell Mol Life Sci* 79: 112
