# Supplementary material for: LUBAC modulates CBM complex functions downstream of TRAF6 in T cells
Source: Nat Commun. 2025 Nov 10;16:9899. doi: 10.1038/s41467-025-65879-6 (PMC12602705; doi:10.1038/s41467-025-65879-6)
Supplement: Supplementary file 2 — Reporting Summary [file 41467_2025_65879_MOESM2_ESM.pdf]

Corresponding author(s): Daniel Krappmann
Last updated by author(s): Oct 14, 2025

# Reporting Summary

Nature Portfolio wishes to improve the reproducibility of the work that we publish. This form provides structure for consistency and transparency in reporting. For further information on Nature Portfolio policies, see our Editorial Policies and the Editorial Policy Checklist.

## Statistics

For all statistical analyses, confirm that the following items are present in the figure legend, table legend, main text, or Methods section.

| n/a | Confirmed | |
|---|---|---|
| ☐ | ☒ | The exact sample size (*n*) for each experimental group/condition, given as a discrete number and unit of measurement |
| ☐ | ☒ | A statement on whether measurements were taken from distinct samples or whether the same sample was measured repeatedly |
| ☐ | ☒ | The statistical test(s) used AND whether they are one- or two-sided<br>*Only common tests should be described solely by name; describe more complex techniques in the Methods section.* |
| ☐ | ☒ | A description of all covariates tested |
| ☐ | ☒ | A description of any assumptions or corrections, such as tests of normality and adjustment for multiple comparisons |
| ☐ | ☒ | A full description of the statistical parameters including central tendency (e.g. means) or other basic estimates (e.g. regression coefficient) AND variation (e.g. standard deviation) or associated estimates of uncertainty (e.g. confidence intervals) |
| ☐ | ☒ | For null hypothesis testing, the test statistic (e.g. *F*, *t*, *r*) with confidence intervals, effect sizes, degrees of freedom and *P* value noted<br>*Give P values as exact values whenever suitable.* |
| ☒ | ☐ | For Bayesian analysis, information on the choice of priors and Markov chain Monte Carlo settings |
| ☐ | ☒ | For hierarchical and complex designs, identification of the appropriate level for tests and full reporting of outcomes |
| ☒ | ☐ | Estimates of effect sizes (e.g. Cohen's *d*, Pearson's *r*), indicating how they were calculated |

*Our web collection on statistics for biologists contains articles on many of the points above.*

## Software and code

Policy information about availability of computer code

| | |
|---|---|
| Data collection | Flow Cytometry: Attune NxT Flow Cytometer (Thermo Fisher Scientific)<br>NovaSeq X Plus (Illumina) for RNA sequencing<br>RT-PCR: Roche LightCycler 480II instrument<br>Cell viability/apoptosis: EnVision 2104 Multilabel plate reader (PerkinElmer) |
| Data analysis | Statistical Analses: GraphPadPrism version 8.0 and 10.0<br>Flow Cytometry: FlowJo software version 10.10<br>RT-PCR: Roche LightCycler 480 software 1.5.1.62 SP3<br>RNAseq: Human genome alignment (GRCh38.p14) by STAR v2.7.11b; Gene-level quantification by Rsubread; Principal component and differential expression analysis using DESeq2; multiple hypothesis testing was corrected using the Benjamini-Hochberg.<br>RNA-sequencing analysis code is available on Zenodo (https://doi.org/10.5281/zenodo.17330166) and GitHub (https://github.com/MendenLab/TRAF6_HOIP_huTcells_RNAseq). |

For manuscripts utilizing custom algorithms or software that are central to the research but not yet described in published literature, software must be made available to editors and reviewers. We strongly encourage code deposition in a community repository (e.g. GitHub). See the Nature Portfolio guidelines for submitting code & software for further information.

# Data

Policy information about availability of data

All manuscripts must include a data availability statement. This statement should provide the following information, where applicable:

- Accession codes, unique identifiers, or web links for publicly available datasets
- A description of any restrictions on data availability
- For clinical datasets or third party data, please ensure that the statement adheres to our policy

The raw numbers for charts and graphs are available in the Source Data file whenever possible. Raw reads (FASTQ) of RNA-sequencing data have been deposited in the Sequence Read Archive (SRA) database under BioProject PRJNA1277211 (https://www.ncbi.nlm.nih.gov/sra/?term=PRJNA1277211). Processed gene-level counts and sample metadata are available in Gene Expression Omnibus (GEO) under GSE299874 (https://www.ncbi.nlm.nih.gov/geo/query/acc.cgi?acc=GSE299874). All data are included in the Supplementary Information or available from the authors, as are unique reagents used in this Article. The raw numbers for charts and graphs are available in the Source Data file whenever possible.

# Research involving human participants, their data, or biological material

Policy information about studies with human participants or human data. See also policy information about sex, gender (identity/presentation), and sexual orientation and race, ethnicity and racism.

| | |
|---|---|
| Reporting on sex and gender | n.a., material received was anonymized with no personal information on sex and gender |
| Reporting on race, ethnicity, or other socially relevant groupings | Healty volunteers from Germany; anonymous recruitment and no records on race, ethnicity or social status were recorded. |
| Population characteristics | n.a., see above |
| Recruitment | Anonymous recruiment of healthy volunteers that provided informed consent. |
| Ethics oversight | Ethical approval was obtained from the Institutional Review Board of the Technical University of Munich (No. 358/15 and 2025-206-S-SB), and the Friedrich Schiller University Jena (No. 2020-1985-Material and 2020-2039-Material). All blood donors provided their informed consent. All work was carried out in accordance with the Declaration of Helsinki for experiments involving humans. |

Note that full information on the approval of the study protocol must also be provided in the manuscript.

# Field-specific reporting

Please select the one below that is the best fit for your research. If you are not sure, read the appropriate sections before making your selection.

[x] Life sciences  [ ] Behavioural & social sciences  [ ] Ecological, evolutionary & environmental sciences

For a reference copy of the document with all sections, see nature.com/documents/nr-reporting-summary-flat.pdf

# Life sciences study design

All studies must disclose on these points even when the disclosure is negative.

| | |
|---|---|
| Sample size | No statistical analysis methods were used to predetermine sample size estimates. For our biochemical and cellular analyses, we lack prior knowledge for effect size estimates. Predetermination of sample size is less relevant for our biochemical and cellular study, because it is a mechanistic rather than a hypothesis-testing study and multiple controls are used. Scientific rigor is ensured by appropriate controls, biological replicates (at least three per experiment) and consistency in complementary but independent approaches (e.g. generation and analyses of independent KO clones, genetic reconstitution, independent assays [such as reporter, biochemical and gene expression analyses]). |
| Data exclusions | No data were exluded from the analyses. |
| Replication | For experiments in primary T cells individual donors are shown seperately and each data point represents a replicate from one donor. Independent results have been obtained from multiple donors, measuring 3-8 replicates for each sample/condition. For cell lines, we in general analyzed more than on KO clone and used genetic reconstitution to confirm important results. Exact numbers of biological replicates are stated for every experiment in figure legends. With one exception, all experiments contain at least 3 independent replicates as indicated in the figure legends. Due to limitation of primary material, unstimulated control of donor B in Suppl. Fig. 1f contains only 2 biological replicates, but we decided to show the data because they confirm results seen in other donors. |
| Randomization | No randomization was performed. Healthy donor blood anonymous donors. |
| Blinding | Blinding was not performed. Blinding was often not applied for technical reasons, because experiments required complex handling procedures. Importantly, blinding was not relevant, because experiments involved objective, quantitative assays (e.g. flow cytometry, RNA-seq, RT-PCR) performed under controlled laboratory conditions and instrument-based objective data acquisition and not analysis subjected to observer interpretation. Replication, standardized procedures and often complementary approaches (see sample size and replication) were |

used to confirm the results in independent experiments.

## Behavioural & social sciences study design

All studies must disclose on these points even when the disclosure is negative.

| | |
|---|---|
| Study description | *Briefly describe the study type including whether data are quantitative, qualitative, or mixed-methods (e.g. qualitative cross-sectional, quantitative experimental, mixed-methods case study).* |
| Research sample | *State the research sample (e.g. Harvard university undergraduates, villagers in rural India) and provide relevant demographic information (e.g. age, sex) and indicate whether the sample is representative. Provide a rationale for the study sample chosen. For studies involving existing datasets, please describe the dataset and source.* |
| Sampling strategy | *Describe the sampling procedure (e.g. random, snowball, stratified, convenience). Describe the statistical methods that were used to predetermine sample size OR if no sample-size calculation was performed, describe how sample sizes were chosen and provide a rationale for why these sample sizes are sufficient. For qualitative data, please indicate whether data saturation was considered, and what criteria were used to decide that no further sampling was needed.* |
| Data collection | *Provide details about the data collection procedure, including the instruments or devices used to record the data (e.g. pen and paper, computer, eye tracker, video or audio equipment) whether anyone was present besides the participant(s) and the researcher, and whether the researcher was blind to experimental condition and/or the study hypothesis during data collection.* |
| Timing | *Indicate the start and stop dates of data collection. If there is a gap between collection periods, state the dates for each sample cohort.* |
| Data exclusions | *If no data were excluded from the analyses, state so OR if data were excluded, provide the exact number of exclusions and the rationale behind them, indicating whether exclusion criteria were pre-established.* |
| Non-participation | *State how many participants dropped out/declined participation and the reason(s) given OR provide response rate OR state that no participants dropped out/declined participation.* |
| Randomization | *If participants were not allocated into experimental groups, state so OR describe how participants were allocated to groups, and if allocation was not random, describe how covariates were controlled.* |

## Ecological, evolutionary & environmental sciences study design

All studies must disclose on these points even when the disclosure is negative.

| | |
|---|---|
| Study description | *Briefly describe the study. For quantitative data include treatment factors and interactions, design structure (e.g. factorial, nested, hierarchical), nature and number of experimental units and replicates.* |
| Research sample | *Describe the research sample (e.g. a group of tagged Passer domesticus, all Stenocereus thurberi within Organ Pipe Cactus National Monument), and provide a rationale for the sample choice. When relevant, describe the organism taxa, source, sex, age range and any manipulations. State what population the sample is meant to represent when applicable. For studies involving existing datasets, describe the data and its source.* |
| Sampling strategy | *Note the sampling procedure. Describe the statistical methods that were used to predetermine sample size OR if no sample-size calculation was performed, describe how sample sizes were chosen and provide a rationale for why these sample sizes are sufficient.* |
| Data collection | *Describe the data collection procedure, including who recorded the data and how.* |
| Timing and spatial scale | *Indicate the start and stop dates of data collection, noting the frequency and periodicity of sampling and providing a rationale for these choices. If there is a gap between collection periods, state the dates for each sample cohort. Specify the spatial scale from which the data are taken* |
| Data exclusions | *If no data were excluded from the analyses, state so OR if data were excluded, describe the exclusions and the rationale behind them, indicating whether exclusion criteria were pre-established.* |
| Reproducibility | *Describe the measures taken to verify the reproducibility of experimental findings. For each experiment, note whether any attempts to repeat the experiment failed OR state that all attempts to repeat the experiment were successful.* |
| Randomization | *Describe how samples/organisms/participants were allocated into groups. If allocation was not random, describe how covariates were controlled. If this is not relevant to your study, explain why.* |
| Blinding | *Describe the extent of blinding used during data acquisition and analysis. If blinding was not possible, describe why OR explain why blinding was not relevant to your study.* |

Did the study involve field work? ☐ Yes ☐ No

# Field work, collection and transport

| | |
|---|---|
| Field conditions | *Describe the study conditions for field work, providing relevant parameters (e.g. temperature, rainfall).* |
| Location | *State the location of the sampling or experiment, providing relevant parameters (e.g. latitude and longitude, elevation, water depth).* |
| Access & import/export | *Describe the efforts you have made to access habitats and to collect and import/export your samples in a responsible manner and in compliance with local, national and international laws, noting any permits that were obtained (give the name of the issuing authority, the date of issue, and any identifying information).* |
| Disturbance | *Describe any disturbance caused by the study and how it was minimized.* |

# Reporting for specific materials, systems and methods

We require information from authors about some types of materials, experimental systems and methods used in many studies. Here, indicate whether each material, system or method listed is relevant to your study. If you are not sure if a list item applies to your research, read the appropriate section before selecting a response.

## Materials & experimental systems

| n/a | Involved in the study | |
|---|---|---|
| ☐ | ☒ | Antibodies |
| ☐ | ☒ | Eukaryotic cell lines |
| ☐ | ☐ | Palaeontology and archaeology |
| ☐ | ☐ | Animals and other organisms |
| ☐ | ☐ | Clinical data |
| ☐ | ☐ | Dual use research of concern |
| ☐ | ☐ | Plants |

## Methods

| n/a | Involved in the study | |
|---|---|---|
| ☐ | ☐ | ChIP-seq |
| ☐ | ☒ | Flow cytometry |
| ☐ | ☐ | MRI-based neuroimaging |

# Antibodies

**Antibodies used**

The following antibodies were used for immunoprecipitation (IP) and Western blot (WB): anti-HA (3F1, HMGU core monoclonal antibodies); anti-CARD11 (1D12, #4435, RRID: AB_10694496), anti-p65 (D14E12, #8242S, RRID: AB_10859369), anti-phospho-p65 (93H1, #3033, RRID: AB_331284), anti-IκBα (L35A5, #4814, RRID: AB_390781), anti p IκBα (5A5, #9246, RRID: AB_2151442), anti-HOIP (E6M5B, #99633, RRID: AB_2891320), anti-SHARPIN (D4P5B, #12541, RRID: AB-2797949), anti JNK1/2 (#9252, RRID: AB_2250373), anti-p JNK (81E11, #4668, RRID: AB_823588), anti-p-ERK (#9101, RRID: AB_331772), anti-Caspase 8 (1C12, #9746, RRID: AB_2275120), anti-Caspase 3 (#9662, RRID: AB_331439), anti-PARP (#9542, RRID: AB_2160739), anti-phospho-RIP1 (Ser166, D1L3S, #65746, RRID: AB_2799693) (all Cell Signaling Technology); anti-BCL10 (H 197, #sc-5611, RRID: AB_634292), anti-BCL10 (C-17, #sc-9560, RRID: AB_2064858, IP: 2.5L), anti-β-Actin (C4, 1:10,000 #sc-47778, RRID: AB_2714189), anti MALT1 (B-12 for human, #sc-46677, RRID: AB_627909), anti-CYLD (E 10, #sc-74435, RRID: AB_1122022), anti-HOIL-1 (H-1, #sc-393753, RRID: N/A) anti-Ubiquitin (P4D1, #sc 8017, RRID: AB_628423), anti-ERK1/2 (C9, #sc-514302, RRID: AB_2571739) (all Santa Cruz Biotechnology); anti-HOIP (#MAB8039, RRID: AB_10676585), anti-Regnase-1 (#MAB7875, 1:500 RRID: N/A) (R&D Systems); anti-SHARPIN (#14626-1-AP 1:5000, RRID: AB_2187734) (Proteintech); anti-TRAF6 (EP591Y) (#ab33915, RRID: AB_778572), anti-BCL10 (EP606Y) (#ab33905, RRID: AB_725640, IP: 1L) (all Abcam); anti-linear ubiquitin (1E3, #ZRB2114, RRID: AB_2938573), anti-FLAG M2 (#F3165, Sigma-Aldrich, WB 1:10,000, IP: 1µl, RRID: AB_259529) (all Sigma-Aldrich); anti-StrepMAB-HRP (#2-1509-001, RRID: AB_3095590) (IBA Gmbh); horseradish peroxidase (HRP)-conjugated secondary antibodies (anti-rabbit (#711-035-152, RRID: AB_10015282), anti-mouse (#711-035-150, RRID: AB_2340770), Jackson ImmunoResearch); All antibodies used for WB and IP were used at 1:1000 dilution if not otherwise stated.

The following antibodies were used for flow cytometry: anti-hCD2-APC (RPA-2.10, #170029-42, eBioscience, RRID: AB_10805740, 1:400), anti-CD3-FITC (#561806, BD Pharmingen, RRID: AB_11154397, 1:100), anti-CD28-APC (17-0289-41, Invitrogen, RRID: AB_10596352, 1:50), anti-human CD4 FITC antibody (RPA-T4, #300506, BioLegend, RRID: AB_314074, 1:200), Ib PE-conjugated (L35A5, #7523S, Cell Signaling, RRID: AB_10950821, 1:100), p-p65 PE-conjugated (pS529) (#558423, BD Biosciences, RRID: AB_647222, 1:100), anti-hTRAF6-AF647 (326019, #FAB3284R, R&D Systems, RRID: AB_3649181, 1:200) and anti-hTCRα/β-APC (#306718, Biolegend, RRID: AB_10612569, 1:200). The following antibodies were used for Image Stream: anti-CD16/32 (114-0161-81, eBioscience, RRID: AB_467132, 1:50) and NF-B p65 XP-Alexa Flour 488-conjugate (D14E12, #49445. Cell Signaling Technology, RRID: AB_2799359, 1:100).

**Validation**

All antibodies are commerically available and were validated for the application and species by their manufacturers, which can be found on the respective webpages. For many antibodies additional validations have been done by us, e.g. loss of detection in KO cells.

# Eukaryotic cell lines

Policy information about cell lines and Sex and Gender in Research

| | |
|---|---|
| Cell line source(s) | Jurkat T cells were obtained from the laboratory of L. Schmitz (University of Giessen) and verified by DSMZ. HEK293 and HEK293T cells were obtained by DSMZ (RRID: CVCL_0045/CVCL_0063). |
| Authentication | Jurkat T cells were authenticated by DSMZ based on STR profiling confirming cell identity. |
| Mycoplasma contamination | Evaluated regularly and all cell lines used were tested negative for mycoplasma contamination. |
| Commonly misidentified lines (See ICLAC register) | No commonly misidentified cell line was used. |

# Palaeontology and Archaeology

| | |
|---|---|
| Specimen provenance | n.a. |
| Specimen deposition | n.a. |
| Dating methods | n.a. |

☐ Tick this box to confirm that the raw and calibrated dates are available in the paper or in Supplementary Information.

| | |
|---|---|
| Ethics oversight | n.a. |

Note that full information on the approval of the study protocol must also be provided in the manuscript.

# Animals and other research organisms

Policy information about studies involving animals; ARRIVE guidelines recommended for reporting animal research, and Sex and Gender in Research

| | |
|---|---|
| Laboratory animals | n.a. |
| Wild animals | n.a. |
| Reporting on sex | n.a. |
| Field-collected samples | n.a. |
| Ethics oversight | n.a. |

Note that full information on the approval of the study protocol must also be provided in the manuscript.

# Clinical data

Policy information about clinical studies

All manuscripts should comply with the ICMJE guidelines for publication of clinical research and a completed CONSORT checklist must be included with all submissions.

| | |
|---|---|
| Clinical trial registration | n.a. |
| Study protocol | n.a. |
| Data collection | n.a. |
| Outcomes | n.a. |

# Dual use research of concern

Policy information about dual use research of concern

## Hazards

Could the accidental, deliberate or reckless misuse of agents or technologies generated in the work, or the application of information presented in the manuscript, pose a threat to:

| No | Yes | |
|---|---|---|
| ☒ | ☐ | Public health |
| ☒ | ☐ | National security |
| ☒ | ☐ | Crops and/or livestock |
| ☒ | ☐ | Ecosystems |
| ☒ | ☐ | Any other significant area |

### Experiments of concern

Does the work involve any of these experiments of concern:

| No | Yes | |
|---|---|---|
| ☒ | ☐ | Demonstrate how to render a vaccine ineffective |
| ☒ | ☐ | Confer resistance to therapeutically useful antibiotics or antiviral agents |
| ☒ | ☐ | Enhance the virulence of a pathogen or render a nonpathogen virulent |
| ☒ | ☐ | Increase transmissibility of a pathogen |
| ☒ | ☐ | Alter the host range of a pathogen |
| ☒ | ☐ | Enable evasion of diagnostic/detection modalities |
| ☒ | ☐ | Enable the weaponization of a biological agent or toxin |
| ☒ | ☐ | Any other potentially harmful combination of experiments and agents |

## Plants

| | |
|---|---|
| Seed stocks | n.a. |
| Novel plant genotypes | n.a. |
| Authentication | n.a. |

## ChIP-seq

### Data deposition

☐ Confirm that both raw and final processed data have been deposited in a public database such as GEO.

☐ Confirm that you have deposited or provided access to graph files (e.g. BED files) for the called peaks.

| | |
|---|---|
| Data access links<br>*May remain private before publication.* | n.a. |
| Files in database submission | n.a. |
| Genome browser session<br>(e.g. UCSC) | n.a. |

### Methodology

| | |
|---|---|
| Replicates | n.a. |
| Sequencing depth | n.a. |
| Antibodies | n.a. |
| Peak calling parameters | n.a. |
| Data quality | n.a. |
| Software | n.a. |

# Flow Cytometry

## Plots

Confirm that:

- [x] The axis labels state the marker and fluorochrome used (e.g. CD4-FITC).
- [x] The axis scales are clearly visible. Include numbers along axes only for bottom left plot of group (a 'group' is an analysis of identical markers).
- [x] All plots are contour plots with outliers or pseudocolor plots.
- [x] A numerical value for number of cells or percentage (with statistics) is provided.

## Methodology

| | |
|---|---|
| Sample preparation | NF-kB signaling analyses: 100,000 cells were seeded and left untreated or treated with TNFa or PMA/Ionomycin for 30 min at 37°C. For IkBa PE staining, cells were washed with FACS buffer and stained for 30 min at 4 °C with Fixable Viability Dye eFluor 780 (1:1000 in FACS buffer, eBioscience). Cells were fixed in 2% PFA (15 min, RT) and permeabilized in IC buffer (0.1 % saponin in PBS) (15 min, RT). Fc block (1:50 in IC buffer, eBioscience) was performed for 7 min at RT. Antibody staining was performed at 4 °C for 30 min in the dark using Ikba PE-conjugated (1:100 in IC buffer). Cells were washed twice with IC buffer, incubated for 15 min at RT, resuspended in FACS buffer and analyzed on Attune NxT Flow Cytometer. For p-p65 staining, cells were harvested after 30 min of stimulation and directly fixed in Cytofix Buffer (BD) for 10 min at 37°C. Cells were washed with FACS buffer and permeabilized in Perm Buffer III (BD) for 30 min on ice. Afterwards, cells were washed with FACS buffer twice and Fc block was added (1:50 in FACS buffer) for 7 min at RT. Staining was performed at 4 °C for 30 min in the dark using p-p65 PE-conjugated (1:100 in FACS buffer). Cells were washed twice with FACS buffer, incubated for 15 min at RT, resuspended in FACS buffer and analyzed on Attune NxT Flow Cytometer. |
| Instrument | Attune NxT Flow Cytometer |
| Software | FlowJo software v.10.10 |
| Cell population abundance | ~90% purity of human CD4 T cells sorted by MACS as determined by flow cytometry |
| Gating strategy | Gating strategies are shown in Supplementary Figure 9. |

- [ ] Tick this box to confirm that a figure exemplifying the gating strategy is provided in the Supplementary Information.

# Magnetic resonance imaging

## Experimental design

| | |
|---|---|
| Design type | n.a. |
| Design specifications | n.a. |
| Behavioral performance measures | n.a. |

## Acquisition

| | |
|---|---|
| Imaging type(s) | n.a. |
| Field strength | n.a. |
| Sequence & imaging parameters | n.a. |
| Area of acquisition | n.a. |

Diffusion MRI ☐ Used ☐ Not used

## Preprocessing

| | |
|---|---|
| Preprocessing software | n.a. |
| Normalization | n.a. |
| Normalization template | n.a. |
| Noise and artifact removal | n.a. |
| Volume censoring | n.a. |

## Statistical modeling & inference

Model type and settings: n.a.

Effect(s) tested: n.a.

Specify type of analysis: ☐ Whole brain ☐ ROI-based ☐ Both

Statistic type for inference (See Eklund et al. 2016): n.a.

Correction: n.a.

## Models & analysis

| n/a | Involved in the study |
|---|---|
| ☒ | ☐ Functional and/or effective connectivity |
| ☒ | ☐ Graph analysis |
| ☒ | ☐ Multivariate modeling or predictive analysis |

Functional and/or effective connectivity: *Report the measures of dependence used and the model details (e.g. Pearson correlation, partial correlation, mutual information).*

Graph analysis: *Report the dependent variable and connectivity measure, specifying weighted graph or binarized graph, subject- or group-level, and the global and/or node summaries used (e.g. clustering coefficient, efficiency, etc.).*

Multivariate modeling and predictive analysis: *Specify independent variables, features extraction and dimension reduction, model, training and evaluation metrics.*
